# Pandemic 2009 H1N1 virus infection associated with purpuric skin lesions: a case report

**DOI:** 10.1186/1752-1947-5-132

**Published:** 2011-04-01

**Authors:** Rocco Urso, Nazario Bevilacqua, Marco Gentile, Daniele Biagioli, Francesco Nicola Lauria

**Affiliations:** 1 Infectious Respiratory Diseases Unit, Clinic Department, National Institute for Infectious Diseases "L. Spallanzani" (INMI), Rome, Italy; 2 Third Infectious Diseases Unit, Clinic Department, National Institute for Infectious Diseases "L. Spallanzani" (INMI), Rome, Italy

## Abstract

**Introduction:**

The influenza virus infection may be severe in non-immune people. Common complications of influenza virus include upper and lower respiratory tract infections, otitis media, myocarditis, acute respiratory distress syndrome and multi-organ failure. There have been cases of vasculitis following influenza vaccination, and rash and acute purpura may occur in certain viral infections. To the best of our knowledge, there are no reports concerning cases of systemic vasculitis associated with pandemic 2009 (H_1_N_1_) infection.

**Case presentation:**

A 23-year-old Caucasian woman was hospitalized at the "L. Spallanzani" National Institute for Infectious Diseases in Rome, Italy. Clinical and radiological features including laboratory findings of this case are illustrated. Notably, the patient had fever, severe abdominal pain, hematuria, arthritis, and purpuric manifestations associated with a normal platelet count. Nasopharyngeal and rectal swabs revealed pandemic 2009 (H_1_N_1_) virus by reverse-transcriptase-polymerase-chain-reaction assay. Routine laboratory analyses showed elevated inflammatory parameters. The autoimmune panel tests were normal. Steroid therapy associated with oseltamivir achieved an evident and rapid improvement. On day seven the patient chose to leave the hospital against medical advice.

**Conclusion:**

Complications related to influenza infection can be life threatening, particularly in immunocompromised patients. Henoch-Schönlein purpura triggered by the novel influenza virus infection could be an attractive pathogenetic hypothesis. We have discussed both the diagnosis and the challenge of therapy protocols. Steroid therapy is part of the management of severe vasculitis. Our case suggests that steroid therapy associated with antivirals can prevent the risk of further complications such as hemorrhage and multi-organ failure during severe vasculitis, without enhancing the virulence of the influenza virus. The possible role of pandemic 2009 (H_1_N_1_) in the pathogenesis of hemorrhagic manifestations should be further investigated.

## Introduction

No cases of vasculitis were ever reported during the pandemic 2009 H1N1 (2009 H1N1) infection, thus its occurrence can be considered rare with undetermined etiology. Systemic vasculitis has been described as an extremely rare event during influenza infection or following the administration of vaccines [1-4]. In addition, influenza infection has seldom been associated with various autoimmune diseases [5]. Among viral respiratory diseases, adenovirus and enteroviruses were previously identified in patients with the onset of rash and cutaneous vasculitis [2,6]. Pandemic 2009 H1N1 had a broad clinical spectrum. The majority of 2009 H1N1 infections are mild and self limiting; however, since the virus can cause serious respiratory disorders in young people and lead to severe complications in non-immune people, early treatment is recommended [7]. In the spring of 2009, the 2009 H1N1 virus spread rapidly throughout the world. The authors describe a case of systemic vasculitis that occurred in an Australian woman suffering from 2009 H1N1 infection. She was hospitalized at the "L. Spallanzani" National Institute for Infectious Diseases (INMI).

## Case presentation

A 23-year-old Caucasian woman spending her holidays in Italy was admitted to INMI in Rome. The otherwise healthy patient presented with a two-day history of fever, severe abdominal pain, diarrhoea with bloody stools and haemorrhagic skin lesions.

**Figure 1:**
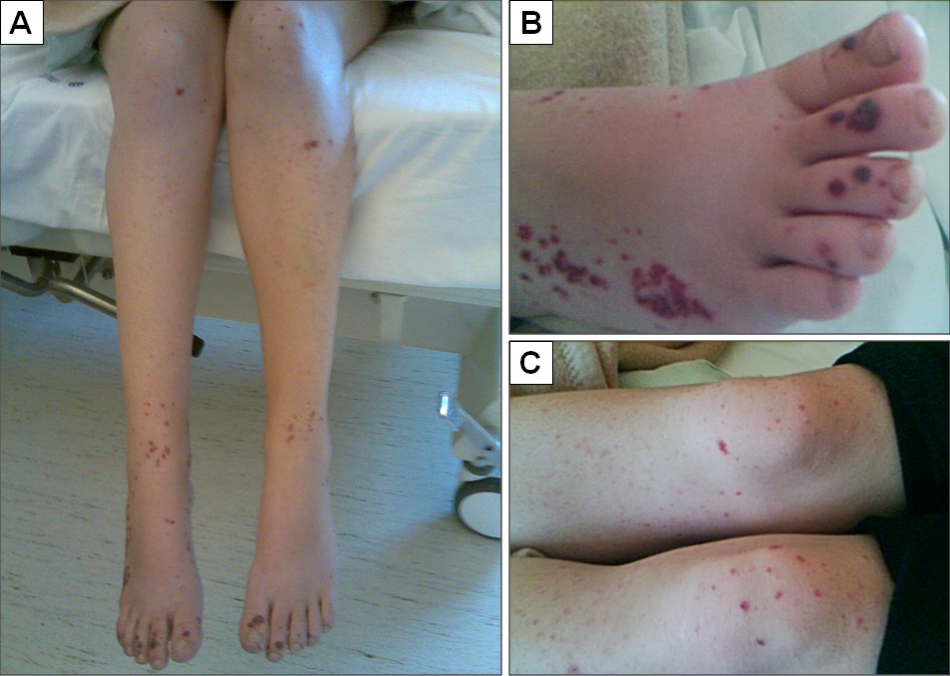
**Overview of the features of purpura before treatment**. **A, C:** show an overview of the features of purpura before treatment. **B**: details the hemorrhagic-necrotic skin lesions on the feet, associated with petechial lesions on the lateral part of the right foot.

Two weeks before, she had rhinorrhoea, a sore throat and a cough. She had received the first dose of human papillomavirus vaccination one month prior to her admittance. On examination, the patient looked pale, tired and feverish with a body temperature of 38.8°C. She manifested an extension of haemorrhagic purpura all over her limbs (Figure 1) and complained of severe abdominal pain. Nasopharyngeal and rectal swabs evidenced a 2009 H1N1 virus by reverse-transcriptase polymerase chain reaction (RT-PCR) assay. Her blood count showed mild anaemia (haemoglobin, 11.70 g/dl), neutrophilia (white blood cells 15.700/mmc: neutrophils 13.400/mmc (85.1%), lymphocytes 1.200/mmc (7,7%), monocytes 800/mmc). C-reactive protein (2.70 mg/dl) and Dimer test values (4.788 ng/dl) were elevated. Platelet count, clotting tests and ESR were normal. A urine examination showed proteinuria, red cells, leukocytes, hyaline and erythrocyte casts. Specific laboratory tests were performed ruling out immunological and autoimmune disorders, including anti-nuclear antibody profile (autoantibodies against RNP/Sm, SS-A, Ro-52, SS-B, Scl-70, Jo-1, centromere B, dsDNA, histones, anti-mitochondrial M2 antibody), anti-smooth muscle, anti-liver-kidney microsomal antibodies, anti-platelets, anti-gastric parietal cell, anti-cardiolipin IgG and IgM, anti-neutrophilic cytoplasmic antibodies (ANCA), lupus anticoagulant, rheumatoid factor (RF), and Coombs test. Serological tests ruled out acute bacterial or viral infections including *Brucella *species, *Salmonella *species, *Rickettsia conori*, *Coxiella burneti *or viruses such as dengue, cytomegalovirus, Epstein-Barr virus, herpes simplex, HIV, coxsackievirus, echovirus and adenovirus. Bacterial nasopharyngeal, blood and urine cultures were sterile. Chest and abdomen X-rays were normal. A computed tomography (CT)-scan of her abdomen and pelvis without contrast revealed only a small effusion in the pouch of Douglas. Oseltamivir (75 mg twice a day, orally) and ciprofloxacin (400 mg twice a day, intravenously) were administered. A short course of methylprednisolone was administered intravenously, starting at 40 mg on July 15 and then reduced by 10 mg daily until it was withdrawn on July 20. Her clinical condition improved during hospitalization. By Day three the leukocytosis decreased (white blood cells 10.100/mmc, neutrophils 5.600/mmc, lymphocytes 3600/mmc). The fever disappeared in three days, her abdominal pain was resolved and there was a marked improvement in the vasculitic lesions. On Day seven she chose to leave the hospital against medical advice.

## Discussion

The rash that can occur during influenza virus infection usually has a macular or maculopapular aspect and can be confused with measles. Hope-Simpson and Higgins [8] found that approximately 8% of influenza B infections and 2% of influenza A virus infections are associated with rash. In addition, Silva and others [1] described a case of a child with a febrile petechial rash associated with influenza A virus infection.

Although cases of vasculitis have occurred following influenza vaccination [3,4], to our knowledge there are no reports concerning cases of systemic vasculitis associated with the 2009 H1N1 infection. An immune thrombocytopenic purpura following human papillomavirus vaccination has been reported [9]. Other vasculitic conditions and sepsis were excluded.

The etiology of Henoch-Schönlein purpura (HSP), which is a systemic vasculitis described mainly in childhood (0.013 to 0.02%), actually remains unknown [10]. In our case, the skin and abdominal haemorrhagic manifestations associated with a normal platelet count could be related to both the 2009 H1N1 infection and HSP. Streptococcal, *Mycoplasma *species, adenovirus, parvovirus and coxsackievirus infections have been reported to precede HSP [10]. In our case, an upper respiratory tract infection occurred first. The main diagnostic hallmarks of HSP include clinical and histological features. In this case the clinical features of HSP such as fever, purpuric rash, bloody stools, hematuria, abdominal pain, joints and kidney involvement, and normal platelet count were present. We did not perform a biopsy of the skin lesions because of the voluntary discharge of the patient.

Vasculitis may result from the interaction between influenza virus antigen A H1N1 and the host's immune response. It has been hypothesized as being a pathogenetic immune-mediated mechanism induced by infections [10,11]. Deregulation of cytokine biosynthesis has been considered the fundamental pathogenetic mechanism related to the severity and progression of 2009 H1N1 influenza [12]. Particularly, the secretion of Th1 and Th17 cytokines was reported as an early host response in severe 2009 H1N1 infection [13] and it is well known that these cytokines are also involved in inflammatory and autoimmune responses [14]. The pro-inflammatory immune response may facilitate the occurrence of vasculitis during a severe pandemic infection.

The spectrum of treatment against vasculitis during influenza infection has been broadly discussed. Steroid therapy was not absolutely contraindicated during 2009 H1N1 infection as reported in the scientific literature. Quispe-Laime and others reported that in patients with H1N1 influenza affected by severe respiratory complications, treatment with a prolonged, moderate dose of corticosteroids was associated with significant improvement in lung injury, multiple organ function scores and low hospital mortality [15]. Carter MJ *et al. *argued that steroids can have an additional role to antiviral therapy in the treatment of severe cases of H5N1 avian flu [13]. In our case, on the basis of a high clinical suspicion of severe vasculitis syndrome and considering the rapid impairment of the clinical conditions, a short-course of steroid therapy was added to the antiviral drugs. Despite the steroid therapy, by Day 6 the nasopharyngeal swab did not show evidence of the presence of 2009 H1N1 virus by RT-PCR. Randomized clinical trials are needed to determine whether additional steroid treatment could be beneficial to dominate spreading of the cytokines, which occurs in severe pandemic H1N1 influenza.

## Conclusion

Systemic vasculitis associated with 2009 H1N1 infection is extremely rare. In this case, the role of the 2009 H1N1 influenza virus in the pathogenesis of hemorrhagic manifestations should be considered. The lack of a histopathological study of the skin lesions may limit our conclusions; however, the macroscopic characteristics of the purpura suggest a vasculitic process. Overall, the clinical, immunological and radiological features are consistent with the clinical diagnosis of HSP. In our case, a short course of steroid therapy in addition to antivirals did not delay the clearance of 2009 H1N1 virus and the combined treatment contributed to improving a complicated case of vasculitis.

## Abbreviations

2009 H1N1: pandemic 2009 H1N1; HSP: Henoch-Schönlein purpura; INMI: "L. Spallanzani" National Institute for Infectious Diseases.

## Consent

Written informed consent was obtained from the patient for the publication of this case report and accompanying images. A copy of the written consent is available for review by the Editor-in-Chief of this journal.

## Competing interests

The authors declare that they have no competing interests.

## Authors' contributions

RU and NB analyzed and interpreted the patient data regarding the infectious issues and were the major contributors in writing the manuscript. All authors read and approved the final manuscript.

## References

[B1] Silva ME Cherry JD Wilton RJ Ghafouri NM Bruckner DA Miller MJ Acute fever and petechial rash associated with influenza A virus infection Clin Infect Dis 1999 29 453 454 10.1086/520240 10476766

[B2] Pagnoux C Cohen P Guillevin L Vasculitides secondary to infections Clin Exp Rheumatol 2006 24 S71 81 16859600

[B3] Patel U Bradley JR Hamilton DV Henoch-Schönlein purpura after influenza vaccination Br Med J (Clin Res Ed) 1988 296 1800 10.1136/bmj.296.6639.1800-b 3136851 PMC2546271

[B4] Tishler M Levi O Amit-Vazina M Immune thrombocytopenic purpura following influenza vaccination Isr Med Assoc J 2006 8 322 323 16805230

[B5] Vilalta Castel E Guerra Vales JM González Gamarra A Abarca Costalago M Alonso Navas F López Pascual JJ Hemolytic anemia caused by cryoagglutinins associated with an influenza A virus infection Rev Clin Esp 1986 179 22 25 3738043

[B6] Costa MM Lisboa M Romeu JC Caldeira J De Queiroz V Henoch-Schőnlein purpura associated with coxsackie - virus B1 infection Clin Rheumatol 1995 14 488 490 10.1007/BF02207691 7586994

[B7] Redelman-Sidi G Sepkowitz KA Huang CK Park S Stiles J Eagan J Perlin DS Pamer EG Kamboj M 2009 H1N1 influenza infection in cancer patients and hematopoietic stem cell transplant recipients J Infect 2010 60 257 263 10.1016/j.jinf.2010.01.009 20138188

[B8] Hope-Simpson RE Higgins PG A respiratory virus study in Great Britain: review and evaluation Prog Med Virol 1969 11 354 407 4313720

[B9] Pugnet G Isebaert L Bagheri H Montastruc JL Laurent G Immune thrombocytopenic purpura following human papilloma virus vaccination Vaccine 2009 27 3690 10.1016/j.vaccine.2009.04.004 19464550

[B10] Tizard EJ Henoch- Schőnlein purpura Arch Dis Child 1999 80 380 383 10.1136/adc.80.4.380 10086951 PMC1717896

[B11] Yang YH Chuang YH Wang LC Huang HY Gershwin ME Chiang BL The immunobiology of Henoch-Schőnlein purpura Autoimmunity Reviews 2008 7 179 184 10.1016/j.autrev.2007.11.012 18190875

[B12] Woo PC Tung ET Chan KH Lau CC Lau SK Yuen KY Cytokine profiles induced by the novel swine-origin influenza A/H1N1 virus: implications for treatment strategies J Infect Dis 2010 201 346 353 10.1086/649785 20030555 PMC7202468

[B13] Carter MJ A rationale for using steroids in the treatment of severe cases of H5N1 avian influenza J Med Microbiol 2007 56 875 883 (**Review**). 10.1099/jmm.0.47124-0 17577050

[B14] Bermejo-Martin JF Ortiz de Lejarazu R Pumarola T Rello J Almansa R Ramírez P Martin-Loeches I Varillas D Gallegos MC Serón C Micheloud D Gomez JM Tenorio-Abreu A Ramos MJ Molina ML Huidobro S Sanchez E Gordón M Fernández V Del Castillo A Marcos MA Villanueva B López CJ Rodríguez-Domínguez M Galan JC Cantón R Lietor A Rojo S Eiros JM Hinojosa C Gonzalez I Th1 and Th17 hypercytokinemia as early host response signature in severe pandemic influenza Crit Care 2009 13 R201 10.1186/cc8208 20003352 PMC2811892

[B15] Quispe-Laime AM Bracco JD Barberio PA Campagne CG Rolfo VE Umberger R Meduri GU H1N1 influenza A virus-associated acute lung injury: response to combination oseltamivir and prolonged corticosteroid treatment Intensive Care Med 2010 36 33 41 10.1007/s00134-009-1727-6 19924393 PMC7080155

